# Effect of recirculation zones on the ventilation of a public washroom

**DOI:** 10.1063/5.0064337

**Published:** 2021-11-02

**Authors:** Krishnendu Sinha, Mani Shankar Yadav, Utkarsh Verma, Janani Srree Murallidharan, Vivek Kumar

**Affiliations:** 1 Indian Institute of Technology Bombay, Mumbai, India; 2 Ansys India Pvt. Ltd., Pune, India

## Abstract

Air-borne transmission can pose a major risk of infection spread in enclosed spaces. Venting the air out using exhaust fans and ducts is a common approach to mitigate the risk. In this work, we study the air flow set up by an exhaust fan in a typical shared washroom that can be a potential hot spot for COVID-19 transmission. The primary focus is on the regions of recirculating flow that can harbor infectious aerosol for much longer than the well-ventilated parts of the room. Computational fluid dynamics is used to obtain the steady state air flow field, and Lagrangian tracking of particles gives the spatial and temporal distribution of infectious aerosol in the domain. It is found that the washbasin located next to the door is in a prominent recirculation zone, and particles injected in this region take much longer to be evacuated. The ventilation rate is found to be governed by the air residence time in the recirculation zone, and it is much higher than the timescale based on fully mixed reactor model of the room. Increasing the fan flow rate can reduce the ventilation time, but cannot eliminate the recirculation zones in the washroom.

## I. INTRODUCTION

Air-borne transmission is a prominent concern for highly infectious diseases like COVID-19. Tiny droplets and aerosol can be carried in the air flow over a distance, potentially exposing a large number of people to the infection. This is particularly dangerous in indoor spaces, where infectious aerosol can be present in the air for a long duration. Ventilation of public spaces and shared facilities is therefore an active area of study in the context of COVID-19.^1–4^ It is recommended that the air in enclosed spaces is replaced with fresh air at regular intervals. Ventilation and air conditioning systems are rated for number of air changes per hour (ACH), which is based on the volume of the enclosed space and the air flow rate through ducts and fans.^5^ This gives an estimate of the average residence time or the mean age of air in the room.

The actual residence time of air in an enclosed space and the related probability of air-borne transmission depends on the air flow pattern set up by the ventilation system.^4^ Specifically, the location of ducts and vents relative to the geometry of the room plays a key role.^6,7^ The inlet and outlet ports set up an air circulation pattern in a given room, which determines the pattern of aerosol spread from a potential source.^8^ Of particular interest are the recirculation zones formed at the corners of a room, and around obstacles.^3,9^ Such regions of recirculating flow are characterized by low air flow velocity and high residence time. Naturally, infections particles can remain in these pockets, while the other parts of the room are well ventilated. The deposition of aerosol on surfaces is also found to be correlated with the location and size of recirculation zones.^8^

Computational fluid dynamics is a powerful tool to study the air flow pattern in enclosed spaces. In the context of COVID-19, the objective is to analyze how the air flow carries the infectious droplets and aerosol from a potential source to susceptible individuals in the room.^4^ The concentration of droplets in a given region over a period of time is used to assess the probability of infection spread. Several mitigation strategies have also been evaluated, including air filters, air purifiers, opening doors and windows, and enhancing the flow rate of the air conditioning system. It is found that the efficacy of these mitigation measures depends critically on the air flow pattern in the room and the location of the source of infection.^8–10^

There are several studies of the air flow in different types of enclosed spaces. These include passenger vehicles,^1^ classroom,^3,4^ restaurant,^10,11^ health care facilities,^12^ elevators,^13^ and public transport.^6,14^ It is clear that the air flow and aerosol distribution pattern varies from one configuration to another, and with changes in the location of source. Here, we study the air flow pattern and aerosol spread in a washroom setup, with a focus on recirculation zones and their effect on the rate of evacuation of infectious aerosol. Washrooms are usually shared facilities in offices, schools, restaurants, and other public spaces, with a large potential of infection spread. Active use of water in toilet flushing and washbasins can be a major source of droplets. These droplets can rise high into the air,^15,16^ and some of them can remain in the air for significant duration of time.

**FIG. 1.**
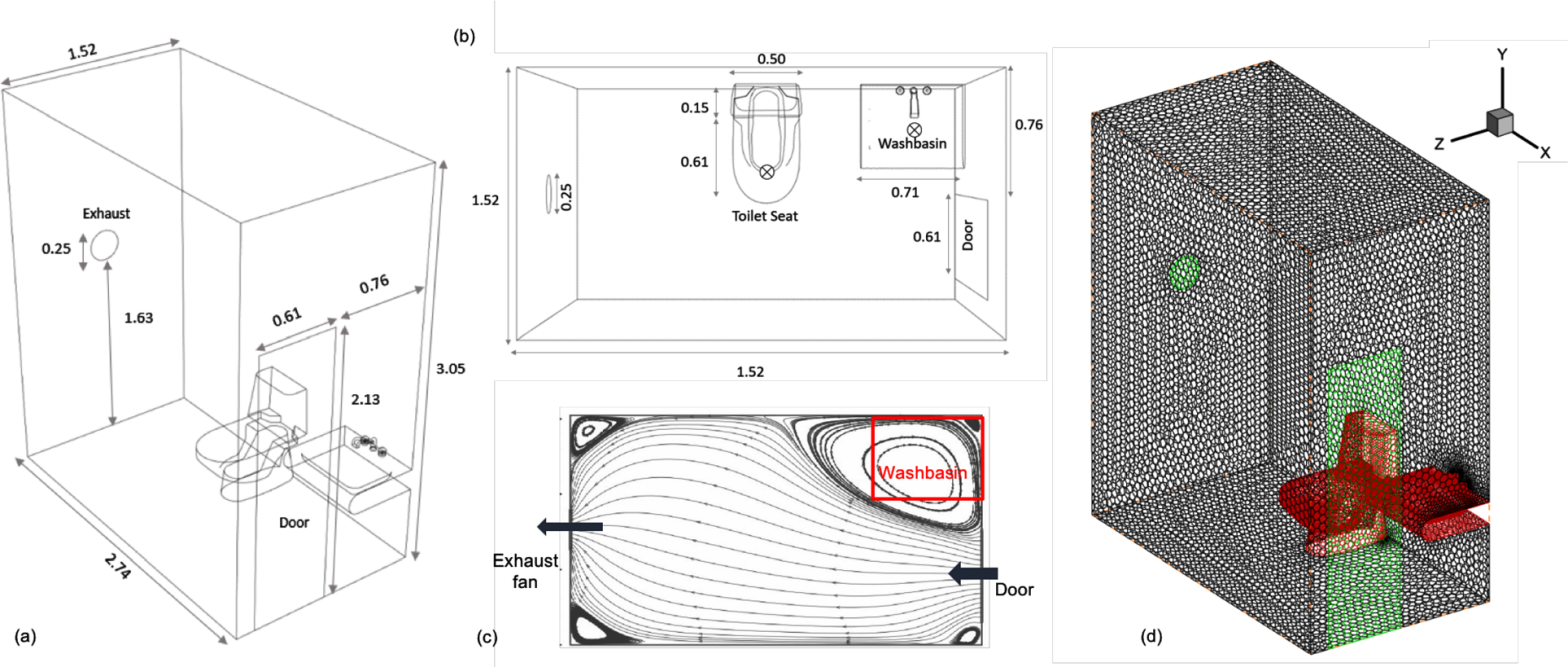
A typical washroom geometry showing an open door, exhaust fan, a washbasin, and a toilet seat: (a) isometric view, (b) top view (c) a typical air flow pattern in top view, and (d) computational mesh for 3D simulation. The particle injection locations are marked by a cross (×) in part (b).

A typical single-person washroom geometry is shown in Fig. 1, with a washbasin next to the door and a toilet seat located in the center of the washroom. Such washrooms are used by multiple people, one after another, and are commonly found in densely populated areas in India. To reduce infection spread from one user to another, it is recommended that the washroom be sanitized after every use. This can be achieved by disinfectant spray and UV-based sanitization. A common practice is to have an exhaust fan evacuate the air out of the washroom. It is recommended that the door be kept open between two consecutive usages of the washroom to increase ventilation. A closed door can hamper the efficacy of the exhaust fan significantly, and the volume flow rate through the fan can be substantially reduced.

The volume flow rate of a typical exhaust fan used in washrooms is 270 m^3^/h, or 158.8 cubic feet per minute (CFM). For the dimensions of the washroom shown in Fig. 1, the fan can vent out an equivalent volume of air in about 170 s. This gives 21.2 air changes per hour (ACH), which means that the fan will refresh the air in the washroom 21.2 times in an hour, or every 170 s. It is based on the assumption that the air in all parts of the room are vented at the same rate. This is not the case in reality. The ACH timescale is also inherent in the mixed reactor model of ventilation,^17^ which assumes that the infectious aerosol is uniformly distributed in the volume of air in the room. CFD simulations in a variety of indoor spaces^9,10^ show that the aerosol distribution primarily depends on the air circulation pattern in the room. Of particular interest are the pockets of air in recirculation zones that can be trapped for much longer than the ACH timescale for a fully mixed reactor model.

The objective of this paper is to use CFD to study the air flow pattern in washrooms, and how it affects the rate at which infectious particles are vented out by the exhaust fan. We specifically identify the regions of recirculating air over the washbasin and near the toilet seat, which can be particularly important in terms of infection spread. The residence time of air in these recirculation zones is compared with that in the primary flow set up between the open door (inlet) and the exhaust fan (exit). We inject particles (simulated droplets and aerosol) in different parts of the washroom and use Lagrangian tracking to quantify their spatial spread in the domain. We also study the time history of their evacuation from the washroom, and compare it with the ACH ventilation rate given by the fully mixed reactor model. Finally, we evaluate the efficacy of a higher fan CFM in venting the particles out of the domain, and eliminating the recirculation zones.

## II. SIMULATION METHODOLOGY

We solve the Reynolds-averaged Navier–Stokes (RANS) equations for incompressible flows using the commercial software ANSYS Fluent (2020 R2 version).^18^ SIMPLE algorithm is used for pressure–velocity coupling, and the realizable k−ε model^19^ is used for turbulence closure. A second-order upwind scheme is used for the convective terms of the RANS equations, whereas a first-order method is employed for the turbulence transport equations. Iterative convergence is achieved when the scaled residual drops below 10^–5^ for the mean and turbulence variables.

Unstructured polyhedral mesh is shown in Fig. 1(d), where the exhaust fan and the door are modeled as outlet and inlet boundaries, respectively. The washbasin and other fixtures are included in the mesh, and dimensions are given in Figs. 1(a) and 1(b). A human is not modeled in the geometry in line with the problem definition, where the ventilation of the washroom is studied between two usages. The mesh consists of $3.2\times 10^{5}$ elements, with a grid resolution of 0.05 m in the interior of the domain. The mesh is much finer near solid boundaries, and the wall-normal spacing corresponds to $y^{+}≃10$ or lower along the walls. The values are relatively higher near the exhaust fan, but it is not expected to affect the rest of the flow field. The flow field results are relatively insensitive to the grid resolution in this range; a coarser grid with 0.1 m interior cell size gives almost identical results for the air flow pattern in the washroom. The variation in velocity magnitude at the center of the washroom is found to be within 1% between the two grids.

The fan CFM is prescribed as an equivalent velocity boundary condition (*V_fan_* = 1.5 m/s) at the exit and the door is specified as a pressure inlet boundary with zero gauge pressure. Rest of the boundaries, including the floor, the ceiling, as well as the surfaces of the washbasin and toilet seat, are prescribed as no-slip viscous wall. Turbulence intensity of 5% is used at the inlet and exit boundaries, along with enhanced wall treatment for the k−ε equations at the no-slip walls.^20^ The inlet value of the turbulent viscosity is prescribed as ten times the dynamic viscosity, which is evaluated at a temperature of 300 K using Sutherland's law. Similar methodology has been applied to air flow simulation in enclosed spaces.^2,3,21,22^

The steady state air flow solution obtained in the domain is used to perform Lagrangian particle tracking. We consider a one-way coupling between the fluid flow and the particles, wherein the effect of the fluid on particles is modeled and the reverse interaction is neglected. The trajectory of the particles is predicted by integrating the force balance on particles,^23^ as per the equation

$$m\frac{d\vec{v}}{dt}=m\vec{g}+{\vec{F}}_{L}+{\vec{F}}_{D},$$

where *m* and $\vec{v}$ are the mass and velocity of a particle and $\vec{g}$ is the acceleration due to gravity.

The lift force ${\vec{F}}_{L}$ is computed using Saffman's model for small particles with particle Reynolds number much smaller than unity.^24^ The model finds application in recent studies of ventilation of enclosed spaces using Lagrangian particle tracking.^3,25^ For particles ranging in diameter from 1 to 5 *μ*, the drag force ${\vec{F}}_{D}$ is given by the spherical drag model,^26^ in which the drag coefficient is formulated for a wide range of Reynolds number.^23,27^ Additional effects due to pressure force and virtual mass force are neglected owing to the small size of the particles.^22^ On the other hand, the particles are assumed to be large enough (greater than 0.5 *μ*) to neglect Brownian forces.^28^ The effect of turbulent dispersion on particle trajectories is modeled using discrete random walk method,^23,29^ where the velocity fluctuations are assumed to be isotropic and follow a Gaussian probability distribution. Similar turbulent dispersion treatment in the context of expiratory aerosols can be found in recent literature.^30,31^

The particles are taken as water droplets of 2.5 *μ* diameter. A mono-dispersed collection is used to study the effect of air flow on particle dynamics. The particle diameter is varied subsequently to study its effect on the ventilation rate. Two injection locations are considered, namely, over the washbasin and on top of the toilet seat. These are frequently used locations, with significant water usage. Toilet flushing can be an important generator of droplets and aerosols.^15^ A total of $3.5\times 10^{5}$ particles are injected over 0.5 s at one of these locations at a height of 1 m, with a nominal injection velocity of 0.1 m/s. These are in the range of values used in similar ventilation studies,^3,8,9,13^ and the sensitivity of the computed solution to these input parameters is reported in Sec. III D. Additionally, we ensure that the number of particles is statistically large enough to get reliable results for particle distribution in different parts of the washroom. The particles are assumed to escape the domain, once they hit a solid wall. The particles may stick to the wall with film formation. However, since our objective is limited to air-borne transmission, and not the contamination of surfaces, we have opted for the escape boundary condition in the majority of simulations presented in the paper. The effect of other boundary conditions in the form of particles getting trapped or reflected from the wall are considered at the end.

Evaporation is not modeled explicitly, given the high variability of evaporation rates with relative humidity and temperature conditions prevailing across different geographical locations. Note that the majority of washrooms in India do not have any form of heating or air conditioning. We are primarily concerned about the air-borne transmission of small droplets and salt nuclei left behind when the droplets evaporate. The particle dispersion timescale (up to 1000 s) is much larger than the evaporation timescale of small droplets (within 1 s for droplets up to 10 *μ* diameter).^32^ Thus, the evaporation kinetics is expected to have negligible effect on the dispersion of the particles.^9,32^

A fluid residence time is calculated based on a user-defined scalar in ANSYS Fluent.^23^ A general scalar convection equation of the form

$$\frac{\partial \rho \phi }{\partial t}+\frac{\partial \rho u_{j}\phi }{\partial x_{j}}=S,$$

is solved numerically for a given flow velocity field *u_j_*, where S=ρ gives ϕ as the residence time of the air. The equation is integrated over the steady state velocity field, with a zero value specified at the inlet boundary. The value of the flow residence time thus obtained gives the time taken for a fluid parcel to traverse the distance along a flow streamline. The higher the value, the longer it takes for a parcel of fresh air to reach a given point from the door inlet. Conversely, low residence time is associated with quick ventilation. A distribution of fluid residence time is used to identify the well-ventilated regions of the washroom, as opposed to the regions of trapped air in recirculation zones or dead-air regions.

## III. RESULTS

### A. Air flow simulation results

**FIG. 2.**
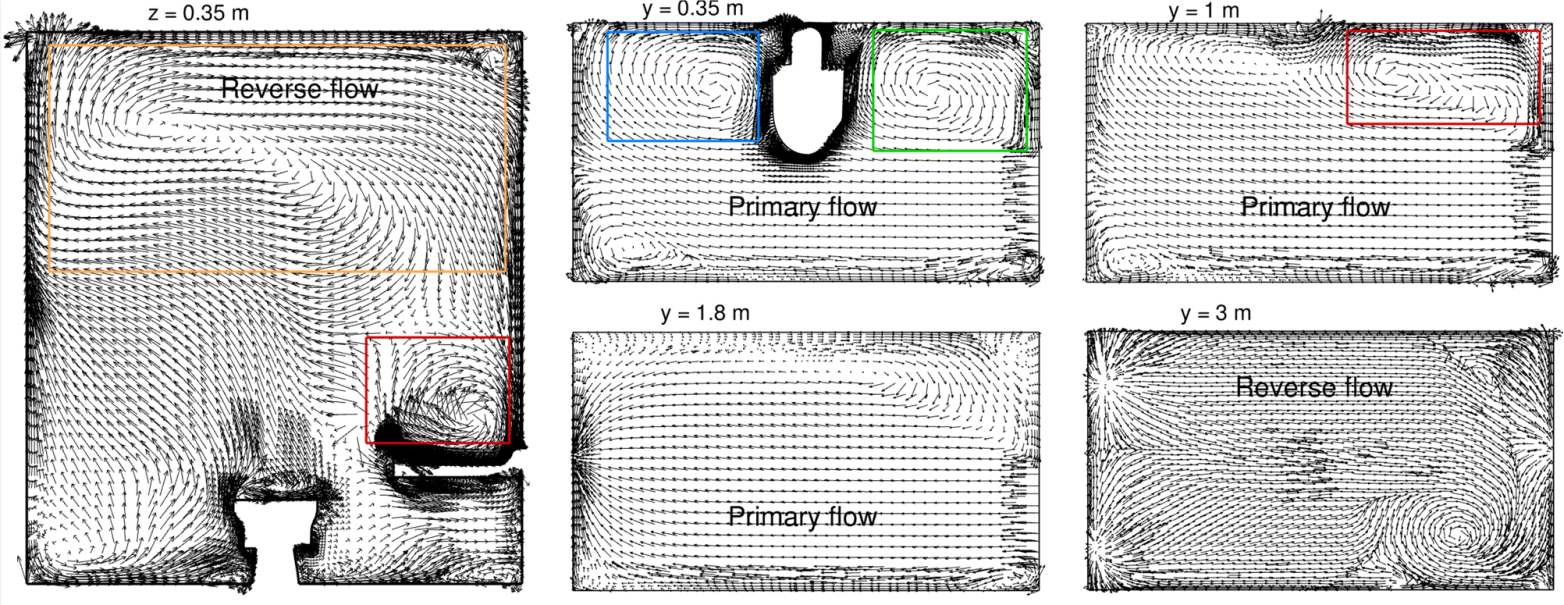
Velocity vectors at different horizontal (top view) and vertical (side view) planes to visualize the three-dimensional flow field computed in the washroom. The recirculating regions over the washbasin (red box), under the washbasin (green box), next to the toilet seat (blue box), and near the ceiling (yellow box) are identified in the plots.

Figure 2 shows the computed flow field solution in terms of velocity vectors in one vertical and several horizontal cross sections. The vertical plane at *z *=* *0.35 m passes through the washbasin and the toilet seat. The horizontal plane at *y *=* *0.35 m is located below the washbasin, *y *=* *1.0 m plane is above the washbasin level, *y *=* *1.8 m passes through the exhaust fan, and *y *=* *3.0 m is close to the ceiling. A primary flow is set up (from right to left) between the door and the exhaust fan, and we see uniform velocity vectors in all the horizontal planes, except for the one near the ceiling. Recirculation regions can be observed either at the corners of the washroom, for example, over and under the washbasin, or around obstacles like the toilet seat. There is a large region of reversed flow (left to right) near the ceiling (at *y *=* *3.0 m), and it is part of the recirculating flow visible in the top part of the vertical plane. We are particularly interested in the recirculation zone formed over the washbasin (marked by a red box) and its role in trapping infectious particles.

**FIG. 3.**
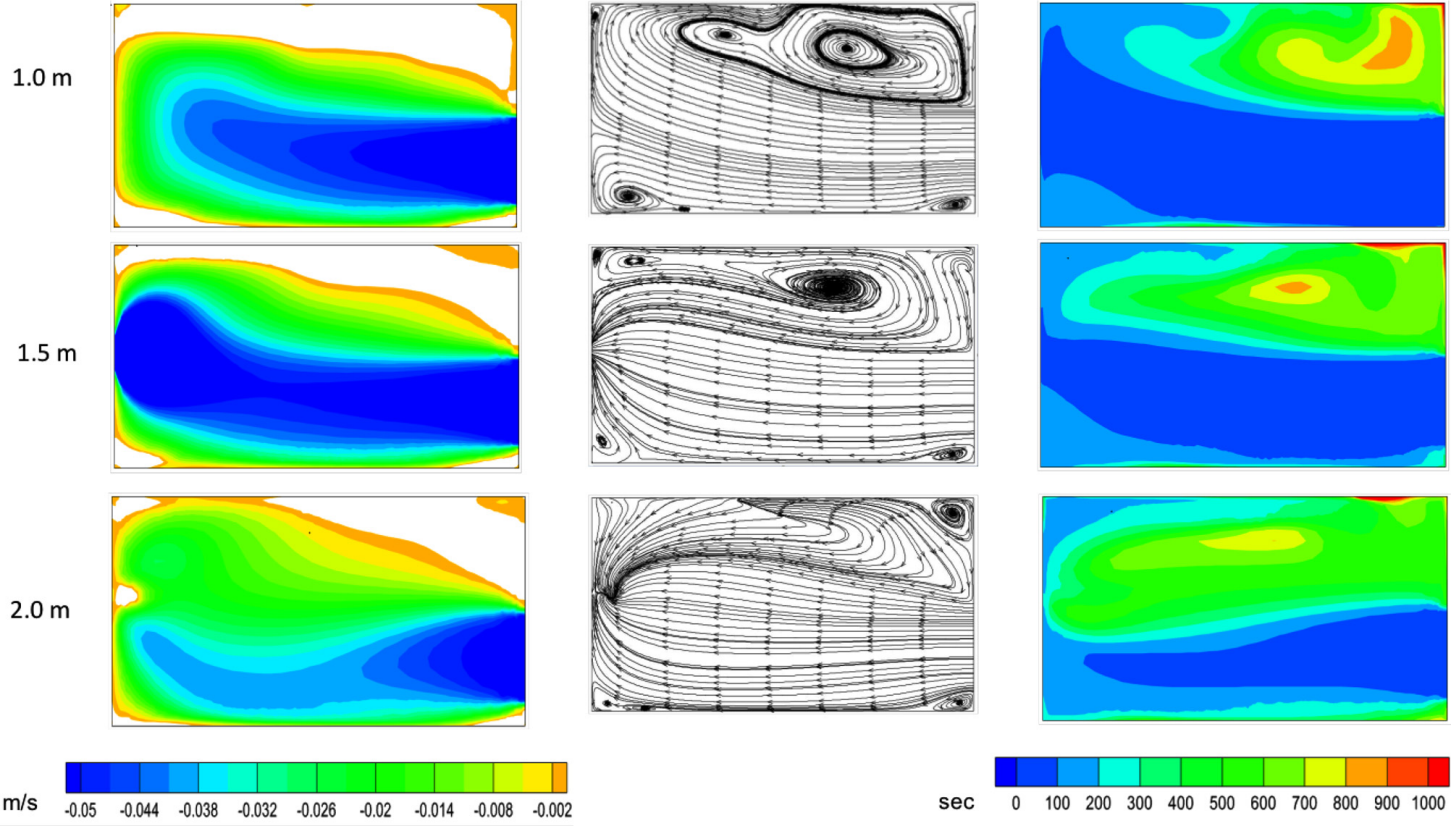
Contours of *x*-velocity (left), streamlines (center), and air residence time (right) plotted in different horizontal sections of the washroom: *y *=* *1 m (top), *y *=* *1.5 m (middle), and *y *=* *2 m (bottom). The recirculation zones are marked by reversed flow (white regions in left panel) and high flow residence time.

Additional details of the three-dimensional flow field are shown in Fig. 3 in terms of *x*-component of velocity, streamlines, and residence time of air. Once again, the data are plotted in three horizontal planes at heights of 1.0, 1.5, and 2.0 m. The residence time of air is calculated based on the procedure described in Sec. II and it gives the mean age of air in different parts of the room. Low residence time in a region implies that it is well-ventilated, while long residence time of air would indicate dead-air zones. The residence time of air also provides a timescale of venting infectious particles from the domain, as discussed subsequently.

The blue region in the velocity contour plots (left panel in Fig. 3) corresponds to the primary flow originating from the door. The magnitude of velocity is relatively high in this region, and it is directed toward the negative *x* axis (from door inlet to fan exit location). The streamlines in the primary flow indicate a direct path from the inlet to the outlet station, and the flow residence time in this region is relatively low (about 50 s). A quick order of magnitude estimate of the time required for air to reach from the door to the exhaust fan can be made as follows. Applying mass conservation between the inlet and outlet locations gives us an approximate air velocity of 5.7 cm/s at the door. This can be assumed to be the characteristic velocity of the primary flow. Taking a ratio of the velocity with the length of the washroom gives the primary flow timescale $\tau_{P}≃$ 50 s. This means that the primary air stream entering the washroom through the door will exit through the fan within this timescale. The primary flow region is thus considered well ventilated.

By comparison, the recirculating regions in the corners are characterized by low velocity, with the white regions in the *x*-velocity plots indicating reversed flow. It is in the positive *x*-direction, opposite to the primary flow. A large region of reversed flow is present over the washbasin and it joins with the recirculating region formed due to the toilet seat to cover the entire length of the washroom. The streamline pattern in *y *=* *1 m cross sections shows imprint of the two vortices. The residence time of air in the recirculating regions is significantly higher than the primary flow, as the streamlines do not have a direct path of exiting the domain. It is in the range of 200–800 s, while a small near-wall region on the top-right corner shows even higher residence time. This is because of a smaller secondary vortex formed in this corner, as indicated by the streamlines in the *y *=* *2 m plane. Similar corner vortex pattern, but to a smaller extent, is also present at lower height (*y *=* *1 and 1.5 m). The thin near-wall region with residence time of 1000 s (red color) is not expected to play a major role in the ventilation of infectious particles in the interior of the washroom.

### B. Particle tracking results

**FIG. 4.**
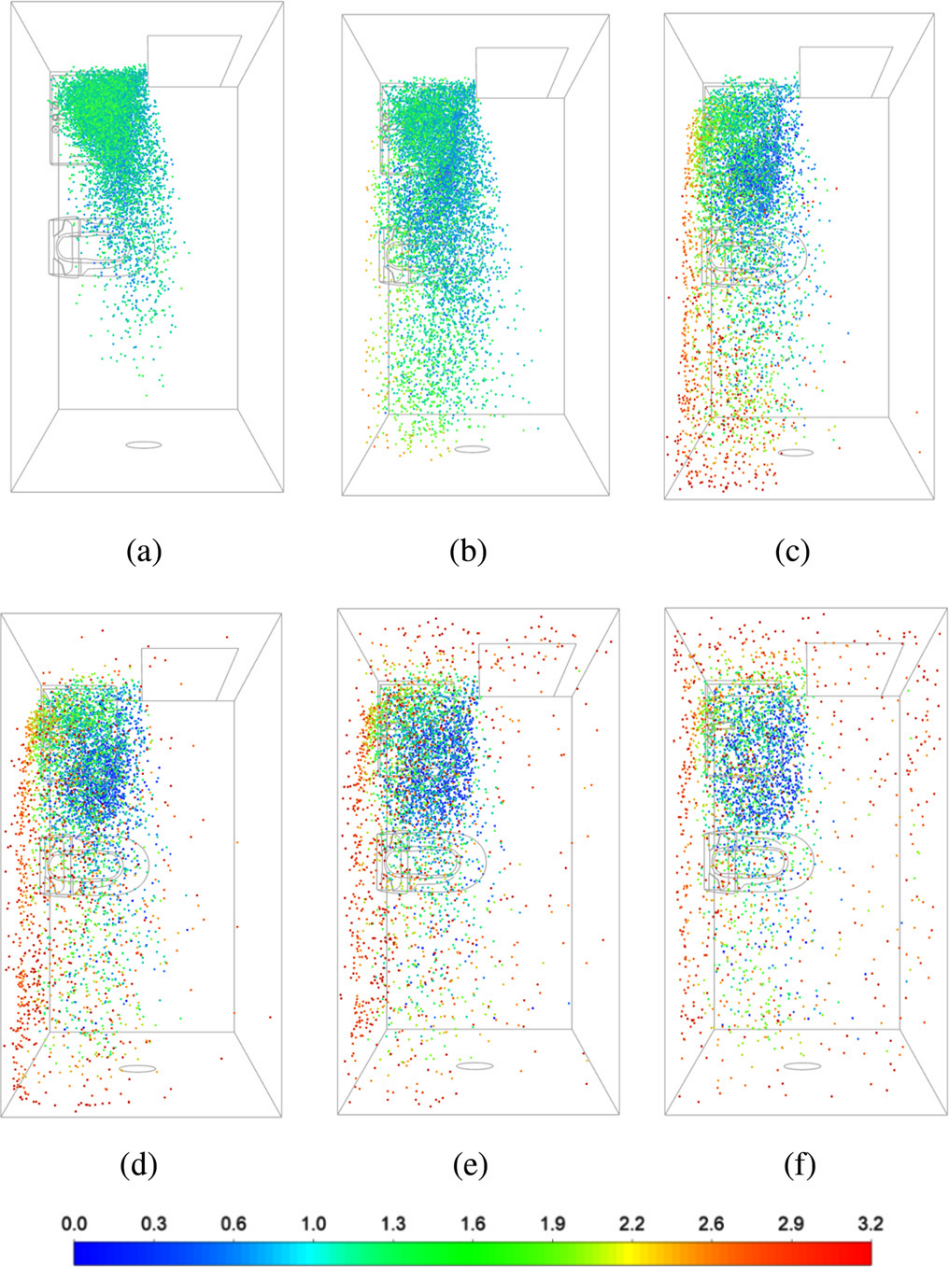
Spatial distribution of particle in the domain at different instants of time measured from the time of injection at the washbasin: (a) *t *=* *50, (b) *t *=* *100, (c) *t *=* *200, (d) *t *=* *300, (e) *t *=* *400, and (f) *t *=* *500 s. The particles are colored based on their height, such that red particles are close to the ceiling, green particles are at the height of a person, and blue particles are close to the floor.

Particles are injected at the washbasin at *t *=* *0 and their locations are tracked over time. The particle tracking simulation details are given in Sec. II. Figure 4 shows the particle distribution in the washroom at different instants of time, from 50 to 500 s. The particles disperse and spread in the domain, and are eventually ejected by the exhaust fan. Some particles, however, remain trapped at the washbasin (green color corresponding to a 1.3 < *y* < 1.9 m), due to the recirculating flow in this region. Similarly, blue particles (for *y* < 0.6 m) are trapped in the vortex formed under the washbasin. At all times, there are more particles in the left half (recirculating flow) than the right half of the washroom (primary flow). Only exception is at large times (*t* > 300 s), when particles tend to accumulate near the ceiling (red particles, *y* > 2.5 m). The majority of the ceiling is in a recirculation region, with reversed flow, as shown in Fig. 2.

**FIG. 5.**
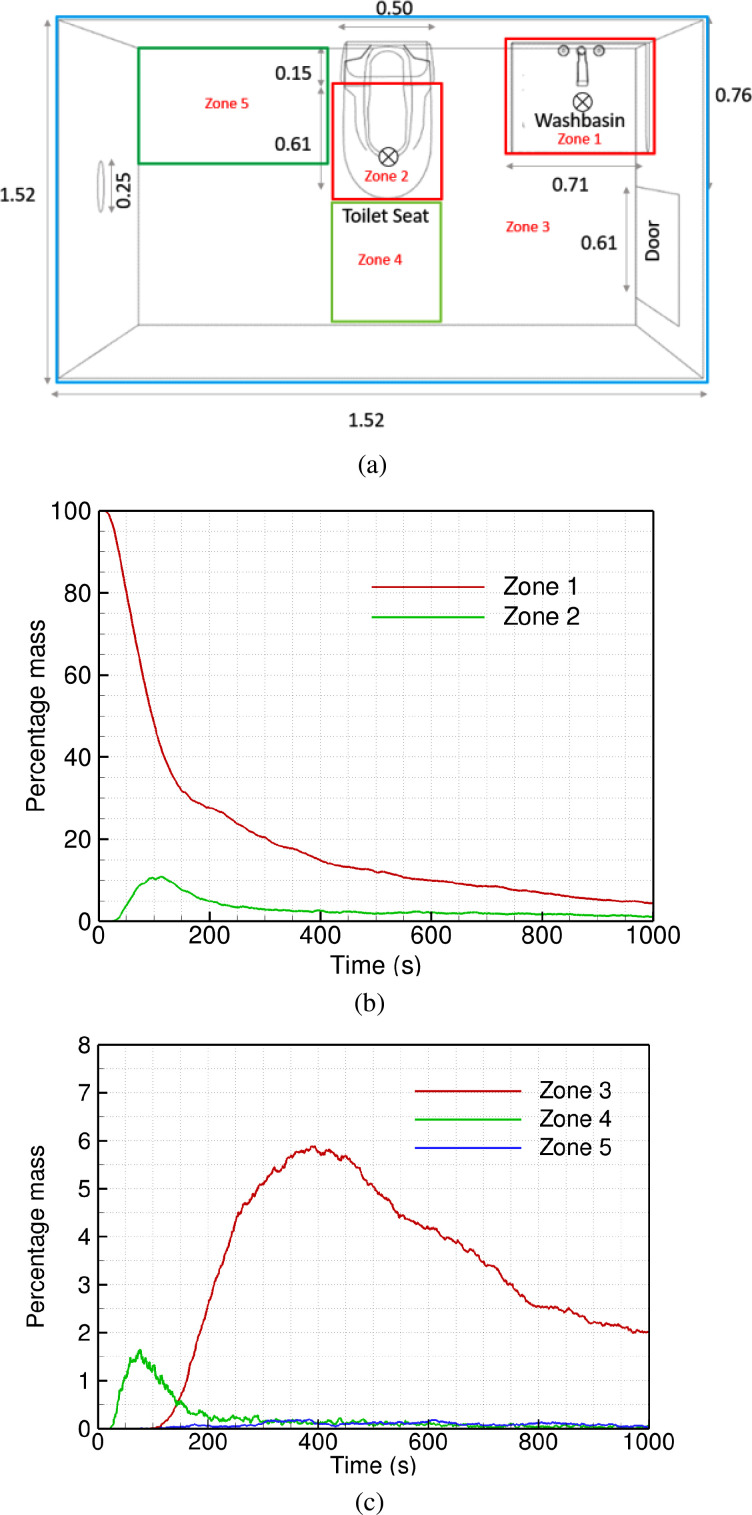
Time history of percentage particle mass, relative to the total mass of particles injected, in different regions of the washroom: (a) different zones; (b) particles in zones 1 and 2; (c) particles in zones 3, 4, and 5.

To study the distribution of the particles in the domain more quantitatively, we define several sub-domains or zones [shown in Fig. 5(a)]. We choose two zones to cover the frequently used areas of the washroom. Zone 1 is in the recirculating region at the washbasin, while zone 2 is over the toilet seat. Zone 3 covers the entire ceiling, above the height of 2.5 m. Zone 4 is in the primary flow between the door and the exhaust fan, and zone 5 is at the corner next to the toilet seat, where we see a second recirculating vortex (in Fig. 2).

The percentage mass of particles in each zone is plotted as a function of time in Fig. 5. It is relative to the total mass of particles injected in the domain at *t *=* *0. Note that all the particles are identical, and there is no change in their mass and size over time due to evaporation. Thus, the percentage mass shown in Fig. 5 is equivalent to the number of particles in each zone relative to the total number injected initially.

As expected, the largest fraction of injected particles is in zone 1; all the particles are injected in this region at t = 0. There is a rapid fall in the particle numbers in zone 1, as the particles disperse in the domain due to turbulent velocity fluctuations. At the same time, there is an increase in the particle numbers in zones 2, 3, and 4. Subsequently, the particle numbers decay in all the zones; the slowest decay is observed in zone 3 at the ceiling. This is in line with the fact that the particles get trapped in the large region of reversed flow at *y* > 2.5 m. On the other hand, there are negligible particles in zone 5 in the corner next to the toilet seat. Overall, at any given time, the number of particles in zone 1 is far greater than the other zones, indicating that the recirculation region formed over the washbasin can trap infectious particles for a long duration of time.

**FIG. 6.**
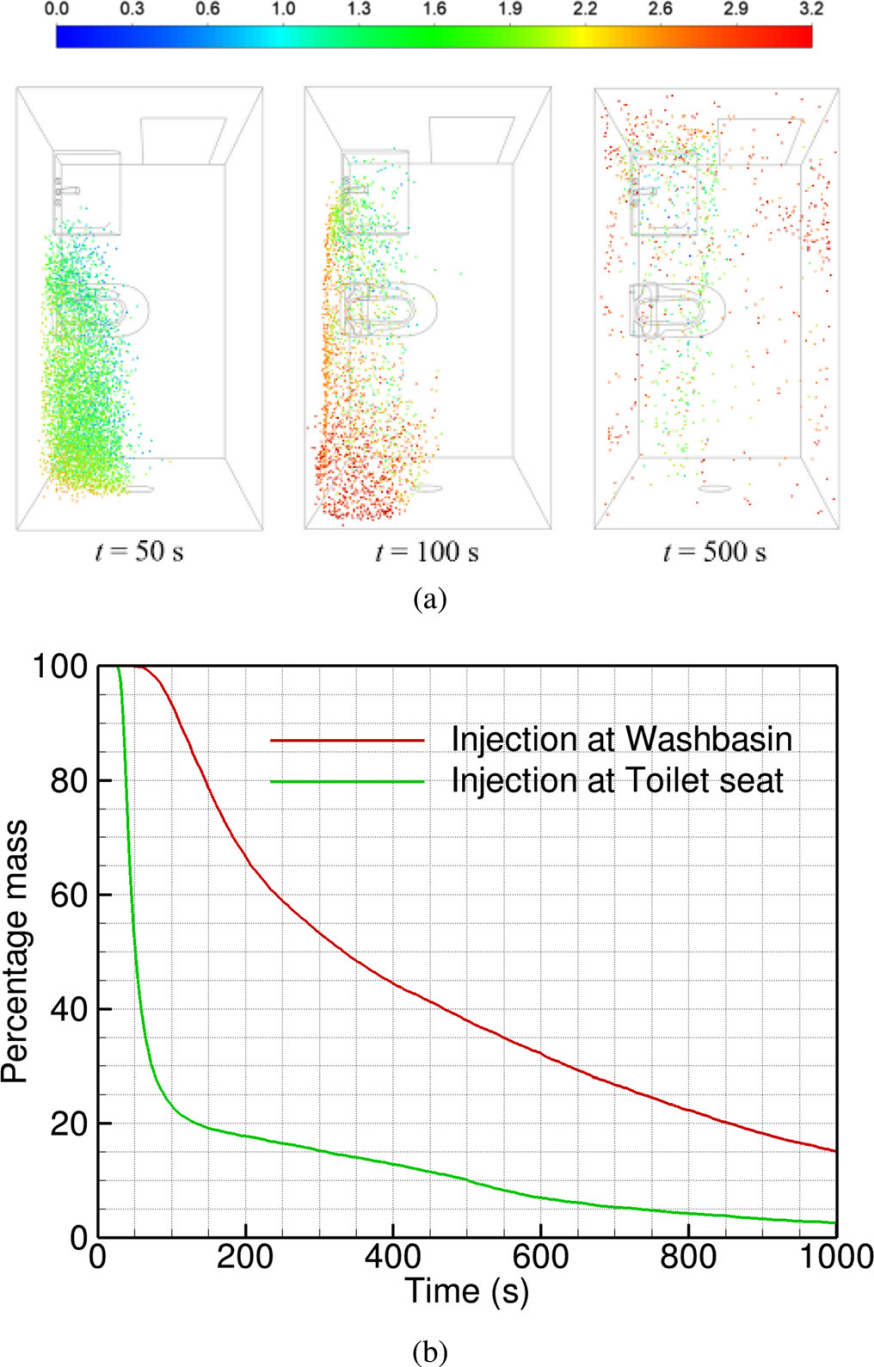
Particles injected at the toilet seat are tracked over time in terms of (a) their spatial distribution and height in m (shown by color), and (b) the mass of particles retained in the domain as a fraction of the initial mass injected at *t *=* *0.

Next, we inject particles over the toilet seat to simulate toilet flushing that generates a large amount of droplets. The toilet seat is located partially in the primary air flow (see Fig. 2) and the particles injected here are directly carried away toward the exhaust fan [see Fig. 6(a)]. The majority of the particles are vented out of the washroom, while some get trapped at the ceiling (red particles with *y* > 2.5 m). There are very few particles present over the washbasin for 1 m <y< 2 m (green particles) at *t *=* *100 s, as compared to the earlier case of washbasin injection (see Fig. 4). This reduces the chances of infection among people present in this frequently used region of the washroom.

The total mass of particles in the domain is plotted as a function of time in Fig. 6(b), for both the injections. It shows a rapid decay for the toilet seat injection, driven by the primary flow timescale of *τ_P_* = 50 s. There is a long tail after the initial drop, possibly because of particles trapped in the recirculating flow at the ceiling level. Only 23% particles are left in the washroom after 100 s, compared to 93% particles in the domain for the case of washbasin injection. In the latter case, it takes about 800 s to bring down the particle number down to 23% of the initial value. Thus, the injection in a recirculation zone results in eight times slower venting of the particles from the domain than injection in primary flow.

### C. Effect of fan CFM

The air flow set up in the washroom is driven by the exhaust fan, and the volume flow rate of air through the fan is expected to play a crucial role. In the simulation, the volume flow rate is specified in terms of the velocity boundary condition prescribed at the fan outlet. We vary the exit velocity from 1 to 3 m/s and study its effect on the air flow pattern and ventilation rate. Note that the fan is rated at 270 m^3^/h, which translates to an exit velocity of 1.5 m/s. A lower exit velocity could be representative of normal wear and tear of the fan, so that it performs at a lower volume flow rate. A higher exit velocity, on the other hand, may be interpreted as increasing the speed of the fan or replacing it with a more powerful fan with a higher CFM.

**FIG. 7.**
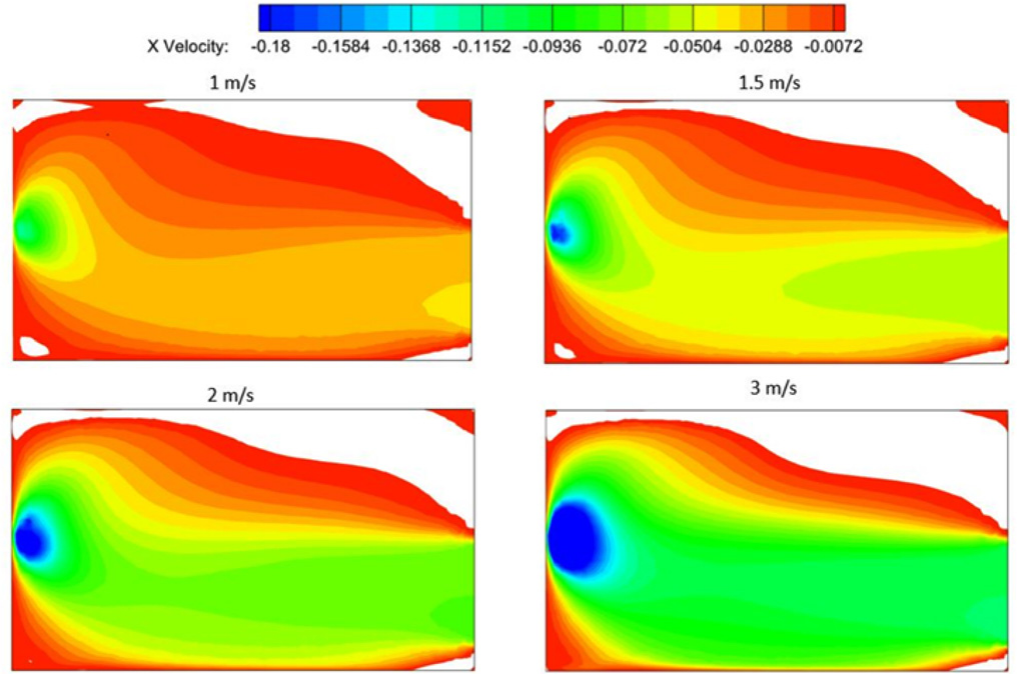
Contours of *x*-component of velocity plotted on a horizontal plane at *y *=* *1.5 m for different values of the fan exit velocity. The white region corresponds to reversed flow (positive velocity), and it gives an indication of the size of the recirculation region at the corners of the washroom.

Figure 7 shows the simulation results for four cases, where the *x*-component of the velocity is used to study the effect of varying the exhaust fan CFM. The data are similar to those presented in Fig. 3, with high negative velocity in the primary flow between the door and the exhaust fan. The magnitude of flow velocity increases proportional to the exit velocity prescribed at the fan, but the qualitative flow pattern remains unaltered by changing the fan CFM. Specifically, the large reversed flow region formed on the washbasin, and marked by white color, is comparable in size and shape between the four solutions. Thus, changing the fan CFM is found to have minimal effect on the size of the reversed flow regions. Increasing the CFM of the exhaust fan further may not be able to eliminate the recirculation zones in the washroom.

**FIG. 8.**
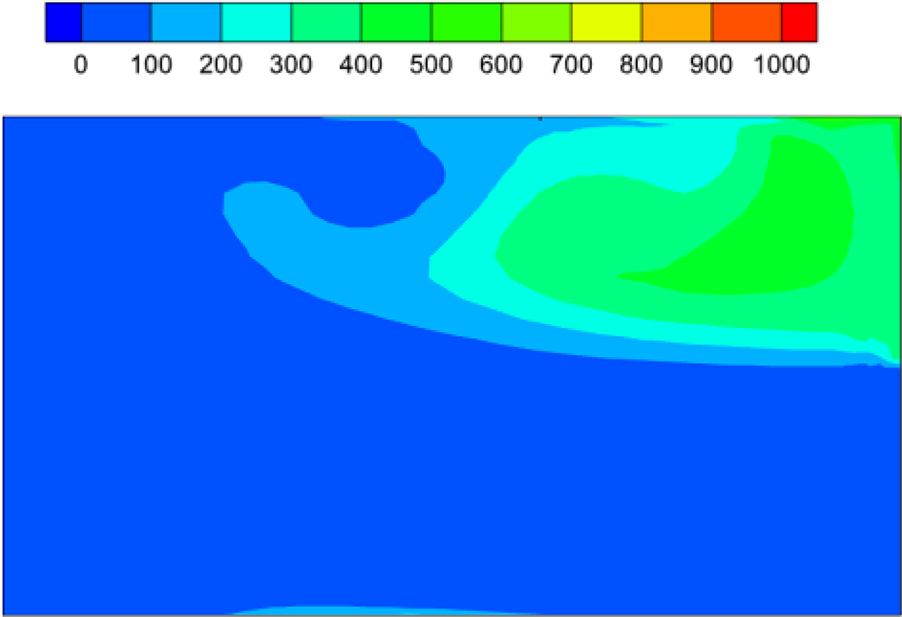
Air residence time (s) contours plotted in a horizontal plane at *y *=* *1.5 m for exhaust fan velocity of 3 m/s. The contour levels are identical to those in Fig. 2 for easy comparison with the residence time for exit velocity of 1.5 m/s.

An increase in flow velocity in the domain, for a higher exhaust fan CFM, decreases the air residence time and vents the used air in the washroom more quickly. Figure 8 plots the distribution of fluid residence time in a horizontal section at *y *=* *1 m, for fan velocity *V_fan_* = 3 m/s and it can be directly compared with the corresponding plot in Fig. 3 for *V_fan_* = 1.5 m/s. The two plots are qualitatively similar, with a higher residence time in the recirculating region, and lower values in the primary flow between the door and the exhaust fan. The residence time in the recirculation region varies between 100 and 400 s for the higher fan CFM, and it is lower than the corresponding values in Fig. 3. The primary flow timescale is reduced by a factor of two, from $\tau_{P}=$ 50 s for the lower fan air velocity to 25 s for the higher exit velocity. This can be interpreted as the time required for air to travel directly from the door to the exhaust fan in the well-ventilated parts of the washroom (primary flow regions marked in Fig. 2), as mentioned in Sec. III A.

Next, we study the effect of the fan CFM on the rate of the decay of particle mass in the washroom. If we assume the washroom as a fully mixed reactor model, we can write the following equation for the particle concentration *C* in the domain:^17^

$$\frac{dC}{dt}=-\frac{Q}{\forall }C,$$

where *Q* is the volume flow rate of air through the fan and ∀ is the volume of the room. We have assumed no evaporation or sedimentation or deactivation. This will give a conservative estimate of the particle concentration in the room as a function of time. For an initial concentration *C*_0_ at *t *=* *0, we have

$$C=C_{0}e^{-t/\tau_{\mathrm{ACH}}},$$

where $\tau_{\mathrm{ACH}}=\forall /Q$ is the ACH timescale based on the volumetric flow rate through the fan. For a fan exit velocity of 1.5 m/s, *τ_ACH_* = 170 s, and it is reduced to 85 s for the 3.0 m/s fan exit velocity.

**FIG. 9.**
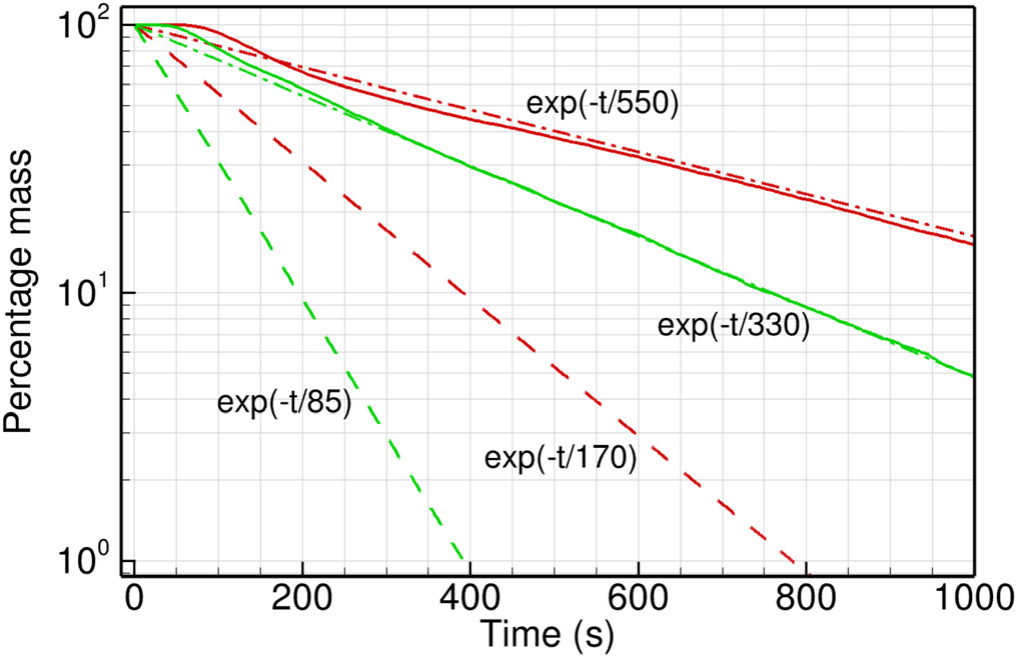
The rate of ventilation as indicated by the decrease in particle mass remaining in the washroom, as a fraction of the total initial mass of particles injected at *t *=* *0. The CFD data (solid lines) for fan exit velocity of 1.5 m/s (red) and 3.0 m/s (green) are compared with the respective theoretical curves (dashed lines) and exponential curve fits (dashed-dotted lines).

Figure 9 compares the decay of particle mass in the domain with the ACH decay rate given above. The percentage of the initial particle mass injected (at *t *=* *0) is plotted for fan exit velocity of 1.5 and 3.0 m/s. For a fixed volume of the domain, the percentage particle mass is equivalent to $C/C_{0}\times 100$ and it is compared to the exponential term $e^{-t/\tau_{\mathrm{ACH}}}$. We have used a logarithmic scale to highlight the exponential trend of the fully mixed reactor model. It is clear from the figure that the particle mass does not follow the ACH decay rate that is frequently used to estimate the ventilation time required for a given volume flow rate. Our results are significantly higher than that predicted by the fully mixed reactor model, implying that assuming the infectious aerosol to be uniformly distributed in the volume of air in the room can be grossly inadequate. This can result in significant under-estimation of the CFM required to achieve a prescribed ACH for a given room.

Interestingly, the CFD data follow an exponential decay with a characteristic time of 550 and 330 s for *V_fan_* = 1.5 and 3.0 m/s, respectively. The values are obtained from an exponential curve fit to the CFD data beyond the initial transient, and are shown as reference lines in the figure. The characteristic decay time is representative of the residence time of the fluid in the recirculation zone (*τ_R_*), as mentioned above. This is true for both the fan CFMs, and it points to the fact that the ventilation process in this scenario is dominated by the recirculating flow. The recirculation zones present in the washroom trap the air and the particles for a long time, delaying the venting of infectious aerosol significantly.

Note that the particle mass decay timescale obtained from CFD simulation is three to four times higher than the ACH timescale. Thus, at any time *t *>* *0, the number of particles in the domain is higher than the estimate of a fully mixed air in the room by a large factor. For example, at *t *=* *500 s, the ACH decay rate with $\tau_{\mathrm{ACH}}=170$ s for a fan exit velocity of 1.5 m/s would predict only 5% particles remain in the domain, while the actual figure is close to 38% in the corresponding CFD data; an increase by a factor of more than seven.

**FIG. 10.**
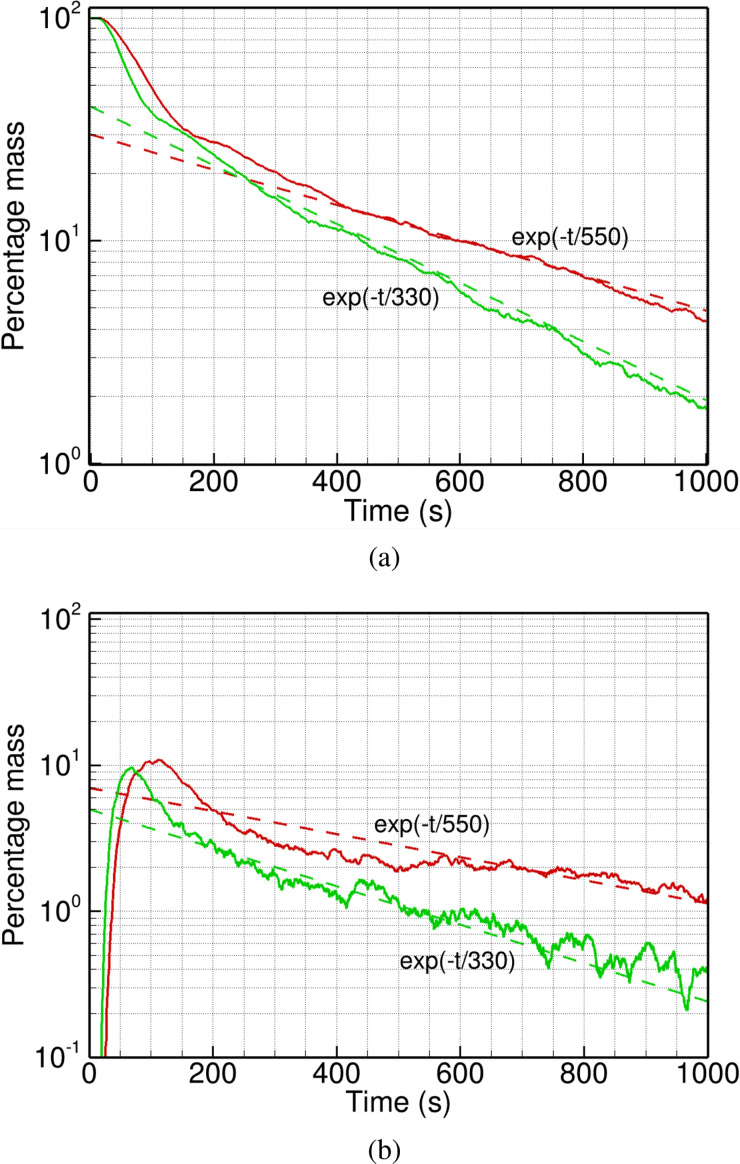
Comparison of the percentage particle mass in (a) zone 1 and (b) zone 2 for fan exit velocity of 1.5 m/s (red) and 3.0 m/s (green). The exponential decay with *τ_R_* = 550 and 330 s, respectively, is also shown for reference (dashed line) and the theoretical curves are offset on the vertical axis to account for the initial transient in each case.

The data presented in Fig. 9 are for injection in the washbasin for both the fan CFMs, as the washbasin can be an important source of droplets and aerosol. The other parameters of particle injection are kept constant between the two simulations. As expected, the time taken to reach a chosen threshold of mass fraction (say 20%) is lower for the more powerful exhaust fan (525 s) compared to the lower CFM case (845 s). In both the cases, the particle mass in the entire domain approximately follows an exponential decay governed by the respective recirculation timescale. The same is true for the particle mass in the two frequently used zones of the washroom, namely, zone 1 above the washbasin and zone 2 over the toilet seat; see Fig. 10. The data computed for zone 1, using *V_fan_* = 1.5 and 3.0 m/s, show a rapid initial drop in particle mass fraction, as the particles disperse out of the washbasin area due to turbulent velocity fluctuations. This is followed by a gradual decay close to the respective recirculation timescale. The two curves are qualitatively similar, except for higher slopes in the higher CFM case.

Zone 2 data in Fig. 10(b) follow a similar trend, except for an initial transient that includes a buildup to a peak value, followed by a drop in particle mass and a relatively steady decay thereafter. Once again, the timescale of the initial transient as well as the subsequent decay are higher for the lower CFM simulation, resulting in a higher mass of particles left in zone 2 than the higher CFM case. The CFD data for *V_fan_* = 1.5 m/s are close to the theoretical decay, while the higher *V_fan_* data are more noisy. This could possibly be caused by the low levels of particle mass in the domain (<1%) for *t* > 500 s in this case. It may require initial injection of more number of particles to get better statistics.

The important point to note is that the ventilation process is qualitatively similar irrespective of the fan CFM, indicating that the flow pattern and the flow processes are not drastically altered by increasing the fan exit velocity. The timescales are reduced by increasing the fan CFM, and this enhances the rate of overall ventilation.

### D. Effect of boundary conditions and injection parameters

**FIG. 11.**
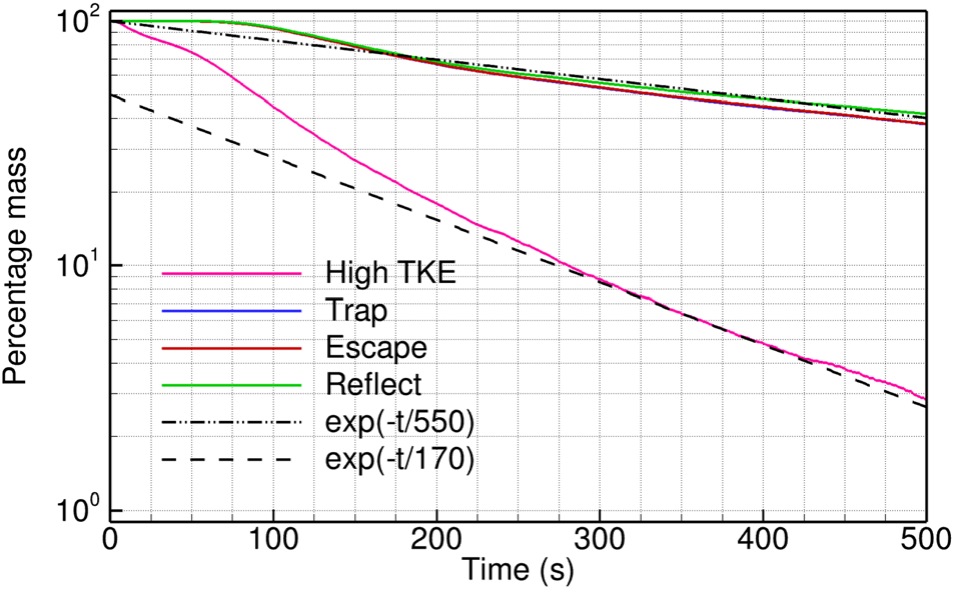
The decay of particle mass in the domain obtained using different boundary conditions: higher turbulence level at inlet boundary and particles reflecting, escaping, or getting trapped at the wall. These are compared with the exponential decay with *τ_ACH_* (dashed line) and *τ_R_* (dashed-dotted line).

We next study the sensitivity of the results presented above to changes in the boundary conditions, both for the air flow simulation as well as the Lagrangian particle tracking. Specifically, we vary the inlet value of the turbulence variables and whether the particles escape, reflect, or get trapped at a solid boundary. All the cases presented in this section are for 1.5 m/s velocity at the exhaust fan, and particle injection at the washbasin. The results are compared in terms of the decay of percentage particle mass in the domain; see Fig. 11.

We note that the earlier results are obtained by specifying a turbulence intensity of 5% and a turbulent-to-dynamic viscosity ratio of 10. These give nominal values for the turbulent kinetic energy (TKE) and the turbulent dissipation rate (*ε*) at the inlet boundary. We compare the percentage particle mass presented earlier in Fig. 9 with that obtained using TKE and *ε* as 1 m^2^/s^2^ and 1 m^2^/s^3^, respectively. These extreme values can be interpreted as high turbulence levels generated at the door inlet due to disturbances in the air flow outside the washroom. It is found that the particle mass fraction decreases more rapidly in the case with higher turbulence level; it falls to 10% at *t *=* *280 s. Interestingly, the percentage particle mass follows an exponential decay with ACH timescale *τ_ACH_* = 170 s for this case.

**FIG. 12.**
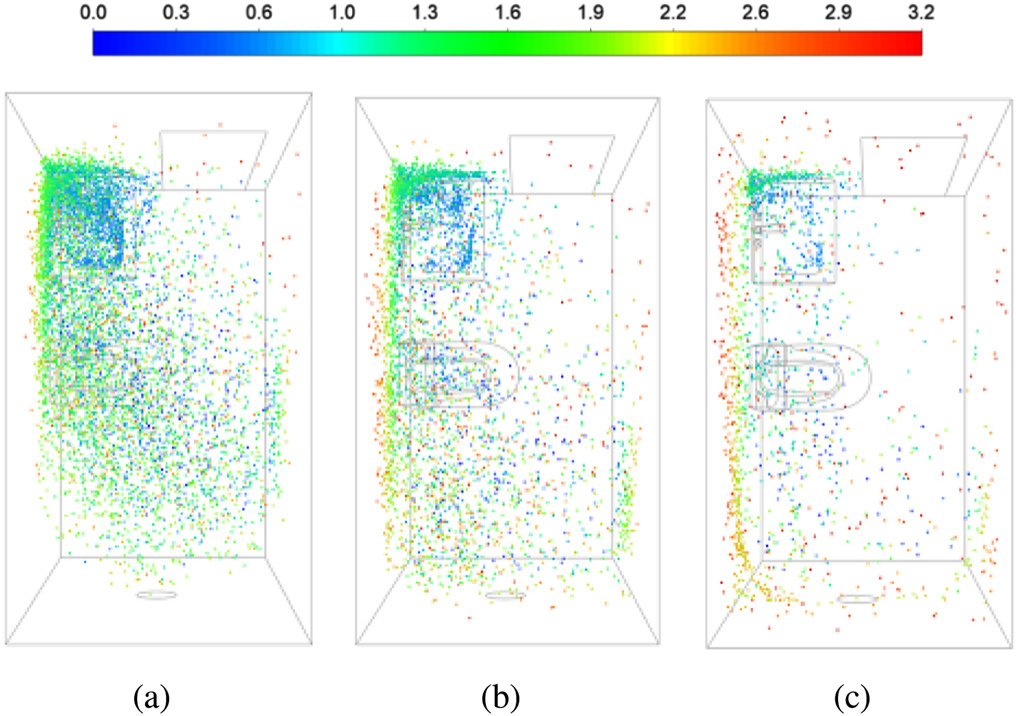
Spatial distribution of particles injected at the washbasin with varying time intervals: (a) *t *=* *50, (b) *t *=* *100, and (c) *t *=* *200 s, computed for the case with high turbulence level. The color legend is identical to Fig. 4 for direct comparison.

The primary effect of varying the turbulence level appears in the rate at which the particles disperse out of the recirculation zone, where they are injected. In the high TKE simulation, the particles spread faster and farther in the room, and it is closer to a well-mixed reactor model of ventilation (Fig. 12). See, for example, the spatial distribution of particles at *t *=* *100 s in comparison to the corresponding time instant in Fig. 4(b). The decay rate of particle mass in the domain (at *τ_ACH_*) supports this observation. The extent of recirculating flow is also altered by the turbulence level in the flow. A higher TKE gives a smaller recirculation zone at the washbasin compared to that shown in Fig. 3 for the lower TKE calculation. A smaller recirculation zone retains a lower number of particles, and this adds to the difference in the observed particle decay time history between the two cases.

The above results indicate that the ventilation rate can be enhanced by increasing the turbulence level in the air flow. This can be achieved by placing additional fans (for example, ceiling fans) to increase the mixing in the room. It can also reduce or disrupt recirculation zones, thereby aiding the exit of particles from the domain. Depending on the level of TKE, the particle decay rate will vary between the timescales *τ_ACH_* and *τ_R_*. A higher turbulence level gets the curve closer to the lower bound, given by the fully mixed reactor model. It is still possible that recirculation zones retain a fraction of the particles (see Fig. 12) and a person placed in such regions is exposed to infection for a much longer time than the rest of the room. A detailed mapping of the room in terms of flow residence time can be used to identify such potentially dangerous locations. Appropriately placed suction or blowing can be used as a retrofit solution to eliminate the recirculation zones.

Figure 11 also shows that the changes in particle decay rate caused by varying the particle boundary condition at solid walls is relatively small. Comparable results are obtained when the particles are assumed to escape from the air flow at the boundary and when they are trapped on the bounding surface. Slightly higher particles mass is predicted when the particles reflect from a solid wall and re-enter the flow domain. The real scenario is expected to be in between the values predicted by the different particle boundary conditions, as a fraction of particles may reflect from a wall, based on their velocity and impact angle. A conservative upper bound can be obtained when all particles reflect from the solid surfaces.

**FIG. 13.**
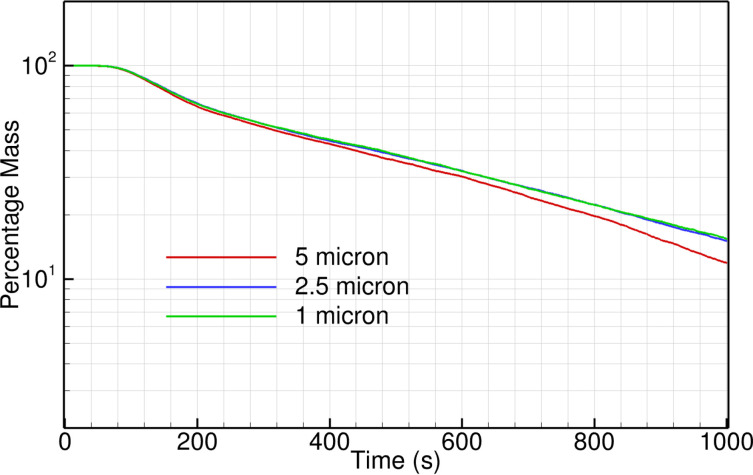
The effect of particle diameter on the decay of particle mass in the domain.

Finally, we study the effect of varying the particle diameter and other injection parameters on the ventilation results. All other parameters are held constant: *V_fan_* = 1.5 m/s, 5% turbulence intensity and escape boundary condition for particles at solid walls. Varying the particle diameter between 1 and 5 *μ* causes minimal changes in the time history of the particle mass in the domain (see Fig. 13). The 1 *μ* results overlap with the 2.5 *μ* data (used in all the earlier simulations), while the 5 *μ* simulation has a slightly lower particle mass in the domain than the smaller diameter cases. This could be because of higher gravitational and inertial effects for the larger particles. The maximum difference between the different diameter simulations is found to be about 3.5% at *t *=* *1000 s.

The difference between the results obtained by varying the droplet diameter could also be interpreted as a margin of error caused by neglecting evaporation. The larger droplets (5 *μ*) would evaporate to a smaller size (1 *μ*) during the course of the simulation, and thus the results presented up to 1000 s are expected to be valid (within 3.5%), even with evaporation, for small particle diameters up to 5 *μ*. Droplets of significantly higher diameter, in the range of 100 *μ*, will fall to the ground much sooner. Sedimentation of large droplets and the resulting surface contamination are beyond the scope of the current study, and will be taken up in the near future.

Table I summarizes the sensitivity of the results to the variation of injection velocity and injection orientation. The percentage particle mass after 400 s is used as a representative value to evaluate the effect on the ventilation rate. The results are very close to the baseline 2.5 *μ* simulation presented in Fig. 13, and the variation is within about ±1% for higher (0.15 m/s) and lower (0.05 m/s) injection velocity, as well as for injection along −*x* and −*y* directions. Note that the baseline simulation has injection velocity of 0.1 m/s along the −*z* direction. Comparatively higher variation is observed when the particle diameter is increased to 5 *μ*, as mentioned above. On the other hand, the lower diameter gives a deviation of only 1.46%. Rosin–Rammler distribution^33^ of particle diameter is also considered, and the results obtained are included in Table I. The distribution has a mean diameter of 2.75 *μ*, a minimum value of 1 *μ,* and a maximum diameter of 5 *μ*. The results are once again close (within 1.15% at *t *=* *400 s) to the baseline simulation.

**TABLE I.** Sensitivity of the computed percentage particle mass in the domain at *t *=* *400 s to the variation of the different input parameters. The sensitivity is quantified as the percentage deviation from the baseline solution.

Case	Percentage mass	Sensitivity
Baseline	44.48	—
Injection velocity = 5 cm/s	44.43	−0.11
Injection velocity = 15 cm/s	44.87	0.88
Injection along −x direction	44.95	1.06
Injection along −y direction	44.91	0.97
Particle diameter = 1 *μ*	45.13	1.46
Particle diameter = 5 *μ*	43.06	−3.19
Rosin–Rammler distribution	44.99	1.15

## IV. CONCLUSION

In this work, we use computational fluid dynamics of air flow and Lagrangian particle tracking to study the ventilation of a public washroom. We consider a single-person washroom that is used by multiple people one after another. The objective is to replace the air in the washroom after every use, so as to vent out any infectious aerosol generated by a user. This is achieved by an exhaust fan that throws air out of the washroom and by keeping the door open between two consecutive usages.

The exhaust fan sets up a primary flow between the door and the exit location, so that the air in this region, along with any infectious particles, is quickly ejected from the washroom within 50 s. There are, however, recirculating flows in the corners and at the ceiling level, which can harbor particles for much longer durations. The frequently used washbasin located next to the door is one such region, and it is of particular interest. Particles injected in the washbasin disperse in the domain and get ejected by the fan, but the rate of ejection is governed by the residence time of air in the recirculating flow. The recirculation timescale of 550 s is about ten times higher than the primary flow timescale. It is also substantially higher than the ACH timescale of 170 s, based on the fully mixed reactor model of the washroom.

Increasing the fan CFM can significantly reduce the ventilation time required to achieve a desired low level of particle concentration in the room. Also, increasing the turbulence level, via additional fans placed inside the washroom, can bring the ventilation rate closer to that of a fully mixed reactor model. However, there can still be pockets of trapped air and higher particle concentration in recirculation zones. It is recommended that such regions are identified in a given room, and frequently used equipment and people are not placed at such locations.

## Data Availability

The data that support the findings of this study are available within the article.
